# Pathology

**Published:** 1879-03

**Authors:** 


### PATHOLOGY.

TRANSPLANTATION OF TISSUES. (*Lancet*, August 24, 1878).

Dr. Zahn has carried out a systematic series of experiments on this subject, first with hyaline and then with fœtal cartilage. The tissues into which the fragments were implanted, were connective tissue, the anterior chamber of the eye, the sub-maxillary glands and the kidneys, testicles and blood vessels. These attempts yielded negative results until fœtal cartilage was employed. If some of the latter were rubbed up with amniotic fluid and injected in the jugular vein, numerous cartilaginous growths were subsequently found in the lungs; and if used in other ways the same effect was produced. The capacity of fœtal cartilage is so great that specimens from the fœtus of a cat gave rise to nodules in the rabbit. Experiments with cartilage from an enchondroma were less successful than the above, but more so than when ordinary adult cartilage was used.

Zahn was also successful with other fœtal tissues. Entire bones were implanted, and although they did not grow as a whole, they preserved their form, and outgrowths occurred. Zahn concludes that only fœtal tissues, or those adult tissues which preserve their fœtal peculiarities, can develop in another part of the same or in another animals. Only, for instance, red marrow will do so, and the periosteum of young individuals.

---

### MATERIA MEDICA AND THERAPEUTICS.

BUTYL CHLORAL. Liebreich. (*Lancet*, July 20, 1878).

It is known that Croton chloral seems to have a selective tendency in its action, affecting first the branches of the fifth pair of nerves.

According to Liebreich, butyl chloral possesses this same peculiarity in a more marked degree. Narcosis may follow very large doses, to be sure, but anæsthesia of the head and face may be complete before any trace of narcosis manifests itself. Liebreich therefore recommends this drug in operations on the face, when other anæsthetics are contra-indicated. For this purpose the dose is 1.0 to 2.0.

While chloral hydrate paralyzes circulation before respiration, the reverse is true with butyl chloral. L. showed this beautifully by opening the thorax in two rabbits, one narcotized with chloral hydrate, the other with butyl chloral. In each case the thorax was opened just as respiration ceased; in the former the heart was lying motionless; in the latter it continued to beat, so long as artificial respiration, which was practiced in both cases, was continued. He thought this property showed the advantage of the drug for purposes of vivisection.

L. refers to the value of the drug in that distressing complaint, tic douloureux, and advises to administer it in glycerine and water, instead of in alcohol, in doses of 1.0 to 2.0.

OIL OF ROSEMARY. Köhler and Schreiber. (*Centralbl. Med. Wiss.*, *No.* 23, 1878.)

These writers have investigated the physiological action of this drug. They find its chief action to be upon the cerebro-spinal nerve centers. The blood pressure falls owing to paralysis of vaso-motor centers in the medulla, but the heart is unaffected; and if the drug is pushed to its utmost there is only retardation of the pulse, while the respiratory center is at last paralyzed. Small doses increase and large doses diminish reflex excitability; repeated hypodermic injections of small amounts have the latter effect. Large doses antagonize the irritability of strychnia poisoning. But its most important action is on temperature, though for this purpose it must be inhaled in a vaporized form, and not given by the stomach. By the former method temperature may be reduced 8°, while by the latter it can not be lowered more than 2° centigrade. These experiments were made in rabbits and other animals.

The writers do not find that on rabbits it has any abortive action. It may excite peristalsis and so produce slight diarrhœa. It seems also to have diuretic properties, and the urine passed after its ingestion has a violaceous odor. Neither muscular irritability nor the size of the pupil are affected by its use.

If its antipyretic effects are certain, and do not differ whether a human subject be the object of experiment, or a rabbit, a most valuable remedy can be added to our armamentarium.

